# Evaluation of Iterative Reconstruction Method and Attenuation Correction in Brain Dopamine Transporter SPECT Using an Anthropomorphic Striatal Phantom

**DOI:** 10.7508/aojnmb.2016.02.003

**Published:** 2016

**Authors:** Akira Maebatake, Ayaka Imamura, Yui Kodera, Yasuo Yamashita, Kazuhiko Himuro, Shingo Baba, Kenta Miwa, Masayuki Sasaki

**Affiliations:** 1 Division of Medical Quantum Sciences, Department of Health Sciences, Graduate School of Medical Sciences, Kyushu University, Fukuoka, Japan; 2 Radiological Science Course, Department of Health Sciences, School of Medicine, Kyushu University, Fukuoka, Japan; 3 Division of Radiology, Department of Medical Technology, Kyushu University Hospital, Fukuoka, Japan; 4 Department of Clinical Radiology, Graduate School of Medical Sciences, Kyushu University, Fukuoka, Japan

**Keywords:** Attenuation correction, Iterative reconstruction, SPECT/CT

## Abstract

**Objective(s):**

The aim of this study was to determine the optimal reconstruction parameters for iterative reconstruction in different devices and collimators for dopamine transporter (DaT) single-photon emission computed tomography (SPECT). The results were compared between filtered back projection (FBP) and different attenuation correction (AC) methods.

**Methods:**

An anthropomorphic striatal phantom was filled with ^123^I solutions at different striatum-to-background radioactivity ratios. Data were acquired using two SPECT/CT devices, equipped with a low-to-medium-energy general-purpose collimator (cameras A-1 and B-1) and a low-energy high-resolution (LEHR) collimator (cameras A-2 and B-2). The SPECT images were once reconstructed by FBP using Chang’s AC and once by ordered subset expectation maximization (OSEM) using both CTAC and Chang’s AC; moreover, scatter correction was performed. OSEM on cameras A-1 and A-2 included resolution recovery (RR). The images were analyzed, using the specific binding ratio (SBR). Regions of interest for the background were placed on both frontal and occipital regions.

**Results:**

The optimal number of iterations and subsets was 10i10s on camera A-1, 10i5s on camera A-2, and 7i6s on cameras B-1 and B-2. The optimal full width at half maximum of the Gaussian filter was 2.5 times the pixel size. In the comparison between FBP and OSEM, the quality was superior on OSEM-reconstructed images, although edge artifacts were observed in cameras A-1 and A-2. The SBR recovery of OSEM was higher than that of FBP on cameras A-1 and A-2, while no significant difference was detected on cameras B-1 and B-2. Good linearity of SBR was observed in all cameras. In the comparison between Chang’s AC and CTAC, a significant correlation was observed on all cameras. The difference in the background region influenced SBR differently in Chang’s AC and CTAC on cameras A-1 and B-1.

**Conclusion:**

Iterative reconstruction improved image quality on all cameras, although edge artifacts were observed in images captured by cameras with RR. The SBR of OSEM with RR was higher than that of FBP, while the SBR of OSEM without RR was equal to that of FBP. Also, the SBR of Chang’s AC varied with different background regions in cameras A-1 and B-1.

## Introduction

Dopamine transporter (DaT) imaging through single-photon emission computed tomography (SPECT) has been used for the diagnosis of neurological and motor disorders (1, 2). DaT SPECT using ^123^I-N-ω-(fluoropropyl)-2β-carbomethoxy-3β-(4-iodophenyl)tropane (^123^I-FP-CIT) has been reported to be useful for diagnosing the early phase of Parkinson’s disease and differentiating it from essential tremor (3-6).

Semi-quantitative indices for DaT SPECT, such as specific binding ratio (SBR), are widely used as diagnostic parameters and have been reported to be helpful in improving the diagnostic accuracy of some motor disorders (7-10). In general, DaT SPECT image quality and quantification are influenced by the applied devices, acquisition parameters, and reconstruction/correction methods (11, 12).

Guidelines on DaT SPECT have recommended the use of both filtered back projection (FBP) and ordered subset expectation maximization (OSEM) for image reconstruction (1, 2). In our previous study, we compared DaT SPECT images reconstructed by FBP and Chang’s attenuation correction (AC) among different devices and collimators, using an anthropomorphic phantom (13). We found that SBR was significantly different among devices and collimators.

Since images reconstructed by OSEM are strongly affected by reconstruction parameters, it is also important to determine the optimal reconstruction parameters. Furthermore, although the guidelines have recommended the application of AC in DaT SPECT imaging (1, 2), the accuracy of AC varies among the applied methods. In this regard, Ishii et al. reported that different AC methods resulted in different patterns on brain perfusion SPECT (14).

The aim of this study was to determine the optimal reconstruction parameters for iterative reconstruction in different devices and collimators in DaT SPECT imaging. DaT SPECT images reconstructed with iterative reconstruction and FBP were compared with those corrected by different AC methods.

## Methods

### Phantoms

A pool phantom (Akita Machine Engineering, Japan) was used to determine AC for Chang’s method (13). The pool phantom was cylindrical (diameter: 16 cm, height: 15 cm, and volume: 3,016 mL) and filled with 14.8 kBq/mL of ^123^I solution.

The evaluation of DaT SPECT images was performed with an anthropomorphic striatal phantom (NMP Business Support Co., Ltd., Hyogo, Japan) (13). This phantom consisted of chambers for bilateral striatum (12.5 mL) and cerebrum (1,180 mL), according to magnetic resonance images of a healthy subject. The bilateral striatum and background of the phantom were filled with different ^123^I solution concentrations (Table 1). Four striatum-to-background radioactivity ratios (S/B ratios: 8.08, 6.03, 4.03, and 3.01) were examined in this study.

**Table 1:** The radioactivity of right

	Striatum		Background

	Right	Left	
Experiment 1	40.4 kBq/mL	20.2 kBq/mL	5.0 kBq/mL

S/B ratio*	8.08	4.03

SBR_true_**	7.08	3.03	

Experiment 2	40.4 kBq/mL	20.2 kBq/mL	6.7 kBq/mL

S/B ratio*	6.03	3.01	

SBR_true_**	5.03	2.01

* Striatum-to-background radioactivity ratio

** Specific binding ratio of true radioactivity

### Imaging protocol

The data were acquired, using two SPECT/CT devices: 1) Symbia T6 (Siemens Healthcare, Erlangen, Germany), equipped with a low-to-medium-energy general-purpose (LMEGP) collimator (camera A-1) and a low-energy high-resolution (LEHR) collimator (camera A-2); and 2) Infinia Hawkeye4 (GE Healthcare, Buckinghamshire, UK), equipped with an extended low-energy general-purpose (ELEGP) collimator (camera B-1) and a LEHR collimator (camera B-2).

Data acquisition was performed six times in the continuous mode with clockwise and counterclockwise rotations for 5 min/180° (rotational radius: 15 cm). A 128×128 acquisition matrix size was applied with 3.3 mm pixel size (1.45 zoom for Symbia T6 and 1.34 zoom for Infinia Hawkeye4) for all acquisitions.

Image reconstruction was performed on a workstation, using Syngo MI Applications (Siemens Healthcare, Erlangen, Germany) and Xeleris2 device (GE Healthcare, Buckinghamshire, UK). The SPECT images were reconstructed, using FBP and OSEM with AC and scatter correction. The reconstruction parameters for FBP were determined in a previous study (13). Briefly, the images were reconstructed, using a ramp filter after pre-processing by a Butterworth filter (Table 2). The OSEM of Symbia was combined with resolution recovery (RR) (Flash 3D), while 2D-OSEM without RR was used in Infinia.

**Table 2:** Reconstruction parameters for DaT SPECT images

		μ-value [/cm]	Butterworth filter [cycle/cm]		

				Order	Cut-off
FBP	Camera A-1	0.12		8	0.40

	Camera A-2	0.08		8	0.36

	Camera B-1	0.12		8	0.44

	Camera B-2	0.12		8	0.44

OSEM		μ-value [/cm]	Iteration, subset	Gaussian filter FWHM [mm]	

	Camera A-1	0.13	10, 10	8.25	

	Camera A-2	0.09	10, 5	8.25	

	Camera B-1	0.12	7, 6	8.25	

	Camera B-2	0.12	7, 6	8.25	

The triple-energy window (TEW) method was used for scatter correction, with main-energy-window width of 159 keV±10%. Sub-windows with 7% width were set right above and below the main window for scatter correction, respectively.

### Specific binding ratio (SBR)

The region of interest (ROI) for striatum was the contour of each striatum on the CT image (13), placed on the slice with maximum count for striatum. For the background, a rectangular ROI (10×10 pixel) was placed on the occipital and frontal regions.

SBR was defined as the ratio of the counts obtained by subtracting the mean count of the background area (C_b_) from the counts of striatum (C_s_) and C_b_:


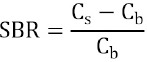


The true SBR (SBR_true_) was calculated using radioactivity in the striatum and background, measured by an auto-well gamma counter (AccuFLEX γ7001, Hitachi Aloka Medical, Ltd., Tokyo, Japan); SBR_true_ was considered as the reference value (Table 1). Considering S/B ratios of 8.08, 6.03, 4.03, and 3.01, SBR_true_ was calculated to be 7.08, 5.03, 3.03, and 2.01, respectively.

SBR using the mean SPECT count of the striatum (C_s, mean_) is referred to as SBR_mean_, whereas SBR using the maximum SPECT count of the striatum (C_S, max_) is denoted by SBR_max_; SBR_SPECT_ consisted of both SBR_mean_ and SBR_max_. The SBR recovery was calculated according to the percentage of SBR_SPECT_ and SBR_true_ as follows:


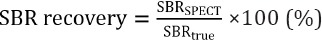


### Determination of reconstruction parameters

The AC in Chang’s method was determined by pool phantom analysis, according to our previous study (13). The number of used iterations and subsets was 10 in Symbia, while eight iterations and five subsets were applied in Infinia. The μ-value of AC varied from 0.06 to 0.15 at 0.01 intervals.

The SPECT image of the pool phantom was evaluated through visual assessment for the flatness of the profile curve, the coefficient of variance (CV), and the summed difference from the reference activity. The flatness of the profile curve was visually classified into five grades by five nuclear medicine physicians (-2: obviously concave, -1: probably concave, 0: flat, +1: probably convex, and +2: obviously convex).

CV was calculated by taking the percentage of standard deviation over the mean activity, using a circular ROI (diameter: 16 cm) on the phantom image. The summed difference from the reference activity was calculated. For this purpose, a rectangular ROI (30×35 pixel) was placed on the reconstruction image. The mean activity of the top two corner pixels was used as the reference value. The difference between the reference activity and each pixel value in the used ROI was summed up (positive: convex; negative: concave). The μ-values were finally determined by evaluating these results comprehensively.

The iteration and subset number of OSEM was determined by evaluating the SPECT images of the anthropomorphic striatal phantom. The iteration number varied from 1 to 10, while the subset numbers were 1, 2, 3, 5, 6, 9, and 10, respectively. At this stage, FWHM of the Gaussian filter was 6.60 mm, which was twice the pixel size.

The striatal phantom images were evaluated by visual assessment, SBR recovery, and CV of the background. On the visual assessment, the clarity of striatum and homogeneity of the background were classified into five grades by a nuclear medicine physician and four radiological technologists (5= excellent, 4= good, 3= normal, 2= poor, and 1= extremely poor).

For SBR recovery, we examined a combination of the iteration and subset number that recovery was converged. A CV of the background lower than 15% was determined as the criterion. After specifying the iteration and subset number, FWHM of the Gaussian filter for OSEM was determined by the visual assessment of the SPECT image, SBR recovery, and CV of the background.

On the visual assessment, the shape, size, and clarity of striatal accumulation and homogeneity of the background were visually classified into five grades by a nuclear medicine physician and four radiological technologists (5= excellent, 4= good, 3= normal, 2=poor, and 1= extremely poor).

### Statistical analysis

The comparison of SBR_SPECT_ was performed by examining SBR recovery and linearity in comparison with SBR_true_. The difference in SBR recovery was analyzed by Tukey-Kramer test. The linearity of the regression line was analyzed by calculating the correlation coefficient between SBR_SPECT_ and SBR_true_. P-value less than 0.05 was considered statistically significant.

## Results

### Determination of reconstruction parameters

The most appropriate μ-value for Chang’s AC was determined to be 0.13 cm^-1^ for camera A-1, 0.09 cm^-1^ for camera A-2, and 0.12 cm^-1^ for cameras B-1 and B-2.

**Figure 1:**
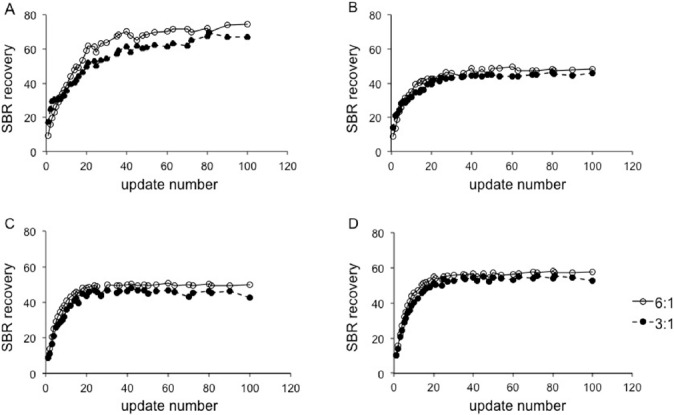
Relationship between the update number and SBR recovery. A) Camera A-1, B) camera A-2, C) camera B-1, and D) camera B-2 (○: SBR=6 and ●: SBR=3). The SBR recovery on camera A-1 converged at update numbers 50 to 100. The other three cameras showed early convergence at update numbers 30 to 50

Figure 1 shows the relationship between the update number and SBR recovery at concentration ratios of six and three. The SBR recovery on camera A-1 demonstrated slow convergence at update numbers 50 to 100, whereas the other three cameras showed early convergence at update numbers 30 to 50.

The images reconstructed using these update numbers were evaluated by visual assessment and CV of the background. According to these assessments, the number of iterations and subsets was determined as follows: 10i10s on camera 1, 10i5s on camera A-2, and 7i6s on cameras B-1 and B-2. These reconstruction parameters were used to evaluate the Gaussian filter.

The optimal FWHM of the Gaussian filter was determined to be 8.25 mm on all cameras, which was 2.5 times the pixel size, since maximum visual score of ≥ 3.5 was obtained with this FWHM in all cameras. The optimal reconstruction parameters determined in this study for each device and collimator are summarized in Table 2. Table 2 also includes parameters for FBP reconstruction, determined in our previous study (13).

### Comparison of DaT SPECT between FBP and OSEM

**Figure 2:**
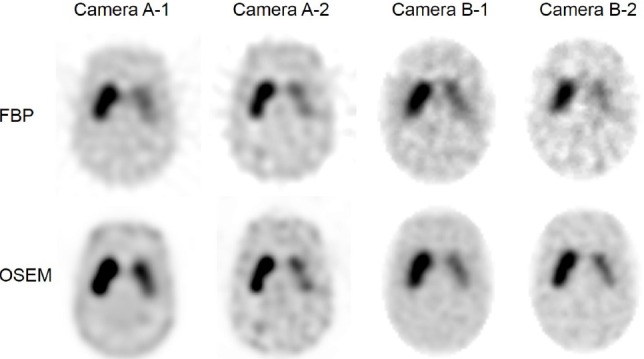
DaT SPECT images. The images in the upper row were reconstructed using FBP and the images in the lower row were reconstructed using OSEM. The background of OSEM images was homogeneous. Edge artifacts were observed on cameras A-1 and A-2, reconstructed using OSEM with RR

Figure 2 shows the SPECT images reconstructed by FBP and OSEM, using the determined reconstruction parameters. Based on the comparison with FBP images, the background of OSEM-reconstructed images was homogeneous. On OSEM images of cameras A-1 and A-2, edge artifacts of high radioactivity delineation were observed. On images acquired by camera B-1, the shape of striatal uptake appeared to be broad and round.

**Figure 3:**
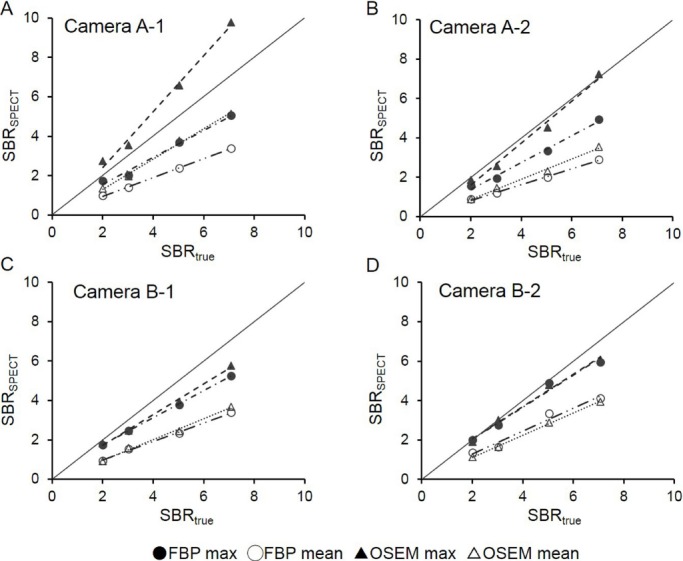
Correlation between SBR_true_ and SBR_SPECT_ in FBP and OSEM reconstruction on each device. A) Camera A-1, B) camera A-2, C) camera B-1, and D) camera B-2. OSEM was superior to FBP in obtaining high recovery. In camera A-1, SBR_max_ of OSEM was overestimated

The recovery of SBR_SPECT_ was compared between FBP and OSEM on each device (Figure 3, & Table 3). The SBR_SPECT_ recovery of OSEM was higher than that of FBP in most cameras. In particular, a significant difference was observed on cameras A-1 and A-2. The highest recoveries for SBR_mean_ and SBR_max_ of OSEM-reconstructed images were 69.7% and 130.6% in camera A-1, respectively; however, SBR_max_ in camera A-1 using OSEM was overestimated (130.6%).

**Table 3:** Linearity and recovery of SBRSPECT for different reconstructions on each device

			SBRtrue				Average	Linearity R2

			2.01	3.03	5.03	7.08		
SBR_mean_	FBP	Camera A-1	49.3%	46.5%	47.3%	47.8%	47.7±1.0%	1.00

		Camera A-2	43.9%	39.2%	39.9%	41.0%	41.0±1.8%	1.00

		Camera B-1	46.8%	51.0%	46.4%	47.9%	48.0±1.8%	0.97

		Camera B-2	67.4%	54.4%	66.4%	58.3%	61.6±5.5%	1.00

	OSEM	Camera A-1	66.9%	64.5%	74.7%	72.7%	69.7±4.2%*┬	0.99

		Camera A-2	43.8%	48.3%	46.0%	50.1%	47.1±2.4%*‡	0.99

		Camera B-1	45.8%	52.7%	48.7%	51.8%	49.7±2.7%	1.00

		Camera B-2	56.3%	54.3%	57.7%	55.7%	56.0±1.2%	0.99

SBR_max_	FBP	Camera A-1	86.0%	66.4%	73.2%	71.4%	74.3±7.2%	0.99

		Camera A-2	77.9%	64.3%	66.2%	70.0%	69.6±5.2%	1.00

		Camera B-1	86.4%	81.6%	75.0%	74.3%	79.3±5.0%	0.98

		Camera B-2	99.2%	90.8%	97.4%	84.2%	92.9±5.9%	0.99

	OSEM	Camera A-1	136.4%	117.0%	130.8%	138.1%	130.6±8.3%*	0.99

		Camera A-2	92.9%	84.6%	89.8%	102.1%	92.4±6.4%*	0.99

		Camera B-1	90.9%	81.3%	78.2%	81.6%	83.0±4.8%	1.00

		Camera B-2	95.1%	99.4%	94.9%	85.9%	93.8±4.9%	0.99

FBP data from (13)

* Significantly higher than FBP (P<0.05),

┬ Significantly higher than cameras 2, 3, and 4,

‡ Significantly lower than camera 4

The second highest recoveries for SBR_mean_ and SBR_max_ were 56.0% and 93.8% in camera B-2, respectively. In terms of SBR_max_, camera A-2 improved using OSEM (92.4%), while use of FBP presented the least favorable result (69.6%). Good linearity of both SBR_max_ and SBR_mean_ was observed on the images of all devices, reconstructed by both FBP and OSEM (R^2^>0.97).

### Comparison of AC methods

**Figure 4:**
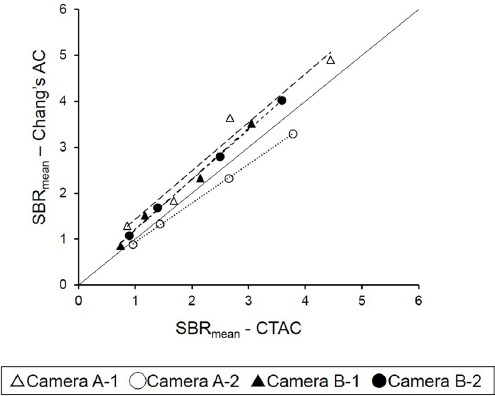
Relationship between CTAC and Chang’s AC regarding SBR_mean_. A significant correlation between SBR_mean_ of Chang’s AC and CTAC was shown on all cameras

The influence of AC was compared between SPECT images corrected by Chang’s AC and CTAC. A significant correlation was observed in all cameras regarding SBR_SPECT_ (Figure 4). SBR with CTAC was slightly smaller, compared to Chang’s AC in camera A-2, while cameras A-1, B-1, and B-2 showed the opposite results; however, the difference was not statistically significant.

Also, the influence of the background region was examined between SBR_SPECT_ calculations, obtained by Chang’ AC and CTAC (Table 4). In Chang’s AC, the ratio of SBR_mean_ obtained in frontal to occipital regions as the background was < 1 on all cameras, while this ratio was > 1 on all cameras in CTAC. The ratio of SBR_mean_ by Chang’s AC was significantly lower than that reported in CTAC on cameras A-1 and B-1.

**Table 4:** The ratio of SBRmean obtained with frontal and occipital regions as the background

2.01		SBRtrue				Average

		3.03	5.03	7.08		
Chang’s AC	Camera A-1	0.91	0.88	0.94	0.91	0.91±0.02*

	Camera A-2	1.01	0.82	1.01	0.86	0.92±0.08

	Camera B-1	0.85	0.90	0.90	0.92	0.89±0.02*

	Camera B-2	0.90	1.05	0.93	1.04	0.98±0.06

CTAC	Camera A-1	1.16	1.03	1.11	1.03	1.08±0.05

	Camera A-2	1.16	0.96	1.11	0.97	1.05±0.08

	Camera B-1	1.03	1.09	1.01	1.06	1.05±0.03

	Camera B-2	1.00	1.06	1.00	1.05	1.03±0.03

* P<0.05 (vs. CTAC)

## Discussion

In this study, we determined the optimal parameters for iterative reconstruction and AC in DaT SPECT images of an anthropomorphic striatal phantom. We found that iterative reconstruction improved image quality and SBR in comparison with the FBP method. The SBR_mean_ calculations in Chang’s AC and CTAC were significantly correlated, while variations were observed depending on the background region in two cameras.

Both SBR_mean_ and SBR_max_ of OSEM reconstruction were higher than FBP reconstruction on cameras A-1 and A-2. The 3D-OSEM of Symbia device (called Flash 3D) incorporated RR, using a point spread function. OSEM with RR was reported to improve the spatial resolution of SPECT images (15).

Oliver et al. reported that SBR obtained by 3D-OSEM reconstruction with RR was higher than that obtained by the FBP method (16). Although RR improves the spatial resolution, the incorporation of resolution modeling in OSEM has been reported to result in edge artifacts (17). It is well known that overestimation due to edge artifacts should be avoided for clinical use.

In the present study, the update number of SBR convergence on camera A-1 was larger than that on other cameras. Overall, a delayed convergence is considered to be an influence of resolution modeling. In this regard, Dickson et al. reported that the update number of convergence was different for each collimator (18).

The incorporation of resolution modeling is considered to delay the convergence of SBR recovery. Therefore, a relatively large iteration number is necessary to reconstruct images using OSEM with resolution modeling. Accordingly, further examination to evaluate overestimation due to edge artifacts is required.

In this study, the SBR recovery of camera A-2 was lower than that of camera A-1. Our previous study also showed the lowest recovery on camera A-2 (13). The collimator used in camera A-2 is basically designed for ^99m^Tc; therefore, it has thin septa and a short hole length (19). The penetration of a high-energy photon from ^123^I should deteriorate the contrast; consequently, camera A-2 is not considered to be appropriate for ^123^I.

On the other hand, SBR_mean_ and SBR_max_ were not different between FBP and OSEM images on cameras B-1 and B-2. Both cameras used 2D-OSEM without resolution modeling. This finding was consistent with a previous study, in which the count concentration of OSEM reconstruction without resolution modeling was similar to that of the FBP method (18). In consistence with the FBP results, the SBR_mean_ and SBR_max_ of camera B-2 were higher than camera B-1 using OSEM reconstruction (13).

The use of CTAC and Chang’s AC did not lead to a significant difference in SBR_mean_ in the present study. Matgorzata et al. reported that the relative uptake of striatum to non-specific uptake in DaT SPECT images, reconstructed with iterative reconstruction and CTAC, was higher than that in images reconstructed by FBP and Chang’s AC (20).

In the mentioned study, the difference in relative striatal uptake between OSEM reconstruction with CTAC and FBP method with Chang’s AC was 27% in normal images and 22% in abnormal images. This difference could not be associated with AC, since reconstruction methods in their study also varied.

According to a previous study, the comparison between AC methods using the same reconstruction method did not show a significant difference on the visual evaluation (21). In this study, camera A-2 showed that SBR with Chang’s AC was smaller than that with CTAC, while other cameras showed the opposite results. Although the difference was not statistically significant, the relatively small μ-value for Chang’s AC in camera A-2 seems to have resulted in this difference due to the large amount of penetration.

On the other hand, in the present study, use of different background regions resulted in different SBRs between reconstructions by CTAC and Chang’s AC. Ishii et al. reported increased frontal and decreased cerebellar count in Chang’s AC in comparison with CTAC (14); this difference is considered to be related to the head holder. Overall, Chang’s AC uses a uniform AC without considering the influence of the head holder. Therefore, SBR_mean_ obtained with the occipital region as the background should be higher than that obtained with the frontal region in Chang’s AC.

This study had several limitations. First, we did not examine the fan beam collimator, which is recommended over parallel-hole collimators due to the advantageous trade-off between resolution and count rate capability (2). Second, this study was a phantom research. Therefore, the reconstruction parameters used for clinical examination should be further evaluated for each device and collimator.

## Conclusion

In conclusion, iterative reconstruction improved the image quality on all cameras, while edge artifacts were observed on cameras with RR. The SBR in OSEM reconstruction with RR was higher than the FBP method, while the SBR of OSEM without RR was equal to FBP. The SBR_mean_ values of Chang’s AC and CTAC were significantly correlated, while the ratios varied with different background regions in cameras A-1 and B-1.
